# A Robust Machine Learning Model for Diabetic Retinopathy Classification

**DOI:** 10.3390/jimaging10010008

**Published:** 2023-12-28

**Authors:** Gigi Tăbăcaru, Simona Moldovanu, Elena Răducan, Marian Barbu

**Affiliations:** 1 Department of Automatic Control and Electrical Engineering, Faculty of Automation, Computers, Electrical, Engineering and Electronics, “Dunarea de Jos” University of Galati, 800008 Galați, Romania; gigi.tabacaru@ugal.ro (G.T.); elena.raducan@ugal.ro (E.R.); marian.barbu@ugal.ro (M.B.); 2 Computer Science and Information Technology, Faculty of Automation, Computers, Electrical Engineering and Electronics, “Dunarea de Jos” University of Galati, 800210 Galati, Romania; 3 The Modelling & Simulation Laboratory, Dunarea de Jos University of Galati, 47 Domneasca Str., 800008 Galati, Romania

**Keywords:** diabetic retinopathy, image processing, entropy, classifiers, machine learning

## Abstract

Ensemble learning is a process that belongs to the artificial intelligence (AI) field. It helps to choose a robust machine learning (ML) model, usually used for data classification. AI has a large connection with image processing and feature classification, and it can also be successfully applied to analyzing fundus eye images. Diabetic retinopathy (DR) is a disease that can cause vision loss and blindness, which, from an imaging point of view, can be shown when screening the eyes. Image processing tools can analyze and extract the features from fundus eye images, and these corroborate with ML classifiers that can perform their classification among different disease classes. The outcomes integrated into automated diagnostic systems can be a real success for physicians and patients. In this study, in the form image processing area, the manipulation of the contrast with the gamma correction parameter was applied because DR affects the blood vessels, and the structure of the eyes becomes disorderly. Therefore, the analysis of the texture with two types of entropies was necessary. Shannon and fuzzy entropies and contrast manipulation led to ten original features used in the classification process. The machine learning library PyCaret performs complex tasks, and the empirical process shows that of the fifteen classifiers, the gradient boosting classifier (GBC) provides the best results. Indeed, the proposed model can classify the DR degrees as normal or severe, achieving an accuracy of 0.929, an F1 score of 0.902, and an area under the curve (AUC) of 0.941. The validation of the selected model with a bootstrap statistical technique was performed. The novelty of the study consists of the extraction of features from preprocessed fundus eye images, their classification, and the manipulation of the contrast in a controlled way.

## 1. Introduction

Diabetic retinopathy (DR) is a common eye disease that, if not treated in its early stages, can affect the quality of life of the patients. In many cases, this illness leads to blindness. When the fundus eye images are analyzed, the ophthalmologists can quantify different stages of DR, non-proliferative diabetic retinopathy, and mild, moderate, and severe stages, according to the severity of each level of the affected blood vessels. If the patient is diagnosed with a high level of DR, he may suffer intraretinal hemorrhages or have definite venous bleeding with prominent intraretinal microvascular abnormalities [1]. DR is asymptomatic in the early stages, so the physicians recommend that patients with diabetes have an annual retinal screening. The examination of the fundus eye is a noninvasive method involving taking a photo of the patient’s eyes with a digital camera.

Following the global statistics, between 2015 and 2019, DR had a prevalence of 27.0% among patients with diabetes. The lowest preponderance was in Southeast Asia at 12.5%, and the highest in the Western Pacific region at 36.2% [2]. From 1990 to 2020, the blindness caused by diabetic eye disease increased from 14.9% to 18.5% [3]. The trend is very worrying, and it shows an increasing number of DR patients, from 382 million in 2018 to 592 million by 2025 [4]. The National Eye Institute provides important information about major eye diseases. It is estimated that 11.3 million people will have DR by 2030, compared with 7.7 million patients nowadays.

Another major problem is premature retinopathy among children from developing countries, with the most affected countries being India, Latin America, Eastern Europe, and China [5]. In benchmarking data provided by the Vermont Oxford Network, the median prevalence of severe premature retinopathy declined from 9% in 2005 to 6% in 2011 [6].

The physicians can diagnose DR with a dilated eye exam, and using drops before the exam, the ophthalmology experts can find abnormalities inside and outside parts of the patient’s eyes. Another advanced procedure is optical coherence tomography (OCT), and this technique consists of obtaining images by scanning the cross-sectional eyes; in this case, the diagnostic is safer [7].

Artificial intelligence tools have a large applicability in medicine; they solve many problems successfully regarding the classification of features extracted from images and the classification of images with convolutional neural networks.

The hands-on engineering methods, such as ensemble learning, deep ensemble networks, and end-to-end learning-based approaches, extract features using advanced or traditional methods [1,8,9]. Ensemble learning algorithms can be used for designing ensembles of neural networks or ensemble machine learning (EL). For creating a model with EL, each selected classifier is trained, the datasets are resampled, and the combination that provides the best classification of variables is kept. The most commonly used for the classification process is the PyCaret tool, which is an open-source library and supervised machine learning module implemented in Python [10].

Our proposal combines image processing, feature extraction, and EL techniques. In the first category, the images are transformed with a non-linear transformation, while the fuzzy and Shannon entropies are computed. Consequently, for the second technique, a large range of machine learning models in a binary classification were proposed. The EL tool was proposed because it mainly includes two parts: the training set, followed by the testing set. Moreover, the ensemble technique supposes bagging, stacking, and boosting voting classifier stages. In the training process, thousands of pieces of data are needed, and the free IDRiD dataset has this potential.

The modification of the contrast is a vital step in image processing applications [11], as in the acquisition stage the images may suffer different artifacts, such as non-uniform illumination or low contrast, and the elimination of this inconvenience with histogram equalization [12] or gamma correction [13] can be performed. The manipulation of the contrast modifies spatial distributions of gray levels so that the extraction of entropy features was completed in preprocessing.

Considering the elements listed above, this paper illustrates original features from preprocessed images, followed by training a robust ensemble learning algorithm. The Shannon and fuzzy features were extracted from fundus eye images when their contrast was manipulated with a gamma correction operation. The transformation of images in controlled ways was performed, and gamma correction belonging to ranges was well established. Section 2 contains related work collected from the scientific literature that deals with DR disease, the typical features, ML tools for the classification process, and accuracy classification. Section 3 discusses the features’ mathematical approaches and the criteria for processing the images. In Section 4, the paper establishes and emphasizes the importance of the tuning process of various ways of ML techniques adopted for the classification of different levels of DR and no DR; this section also contains a discussion of the results, a comparison with other studies, various future directions, and limitations that are proposed to encourage new solutions for early DR detection. Finally, the paper focuses on the conclusions.

## 2. Related Work

Machine learning (ML) learns efficiently from the features and provides successful results if the input data are clear enough. Regardless of the classification of DR, artificial intelligence tools and computer-assisted systems are usually proposed. We compare our study, taking into account the research with and without the tuning processes.

In recent years, various scientific papers have contributed to the DR classification utilizing ML tools. This section presents two main aspects: (i) the features extracted from fundus eye images and (ii) the summarized results from the papers that deal with the ensemble machine learning domain integrated into learning frameworks.

In the following, there are enumerated scientific papers that treat the prediction of diabetic retinopathy levels using various techniques as tuning parameters [14,15], convolutional neural network [16,17], deep ensemble learning (DEL) [1,18,19,20,21], and ensemble machine learning [20,21]. Punctually, the related works are pointed out by references from the following paragraphs [14,15,19,20,21,22,23,24,25].

Assegie et al. [14] developed a hyperparameter-tuned K- nearest neighbors classifier (KNN) model that was based on a dataset that contained 768 instances and 8 features. With this hyperparameter tuning, the accuracy provided by KNN was 82.5%.

Solkar and Das [15] did not use the tuning process on hyperparameters of the support vector machine (SVM) classifier in their study; instead, the classification of features from the APTOS diabetic retinopathy image dataset provided an accuracy of 77.77%.

Ghosh et al. [19] presented pre-trained convolutional neural networks (CNN), such as VGG16 and Inception V3, for improving the classification; the ensemble model was applied to a test set for finding various DR levels, in this way demonstrating the efficacy of the proposed model, and the obtained accuracy and F1 score were 96.4%.

A robust model was pointed out by Nilashi et al. [20], who analyzed the performance of the adaptive neuro-fuzzy inference system. As a result, the classification of features of the retinal images extracted from the Messidor dataset provided an accuracy of 91.5%, a sensitivity of 94.6%, and a specificity of 91.7%.

In 2021, Sikder et al. [21] tested a novel method for DR classification while they were working with the Asia Pacific Tele-Ophthalmology Society in 2019. In the blindness detection (APTOS 2019 BD) dataset, the first features were extracted from an image histogram, and second-order features were extracted from a co-occurrence matrix-fed XGBoost classifier. The presented results show that XGBoost provides the best performance, with an accuracy of 94.20% (margin of error: ±0.32%) and an F-measure of 93.51%.

Antal and Hajdu [22] trained six classifiers as potential members of the ensemble process, and these helped to separate DR and non-DR eye images from the Messidor dataset. The intensity, geometry, and texture features fed the classifiers, and after a binary classification, a sensitivity of 90%, a specificity of 91%, an accuracy of 90%, and an AUC of 0.989 were obtained.

In a recent study, Alshayeji et al. [23] proposed a model able to identify DR levels: normal, mild, moderate, severe, and proliferative. They used an interwoven ensemble learning technique that implied features of a gray-level, co-occurrence matrix correlation, such as homogeneity, entropy, dissimilarity, contrast, and angular second-moment features extracted from Kaggle EyePACS (80,000 images). The model provides an F1 score of 99%, a specificity and sensitivity of 99%, and an AUC of 100%.

The ensemble learning model that included Naive Bayes (NB), K-nearest neighbors, support vector machine (SVM), multilayer perceptron (MP), random forests (RF), and logistic regression (LR) classifiers was proposed by Uppamma and Bhattacharya [24]. For detecting different DR severity levels, the EL model with multidomain features was fed, resulting in the accuracy of the model being 96.5%.

Ramasamy et al. [25] developed a model for the diagnostics of DR, fusing the features extracted from co-occurrence and run-length matrices and the coefficients of the Ridgelet transform features. The performance of the classification with sequential minimal optimization (SMO) was verified. The proposed method was applied to two public datasets, DIARETDB1 and KAGGLE, obtaining an accuracy of 97.05% and 91.0%, respectively.

After reviewing recent papers for eight major state-of-the-art methods [14,15,19,20,21,22,23,24,25], in Table 1, the strengths and weaknesses of our investigations of these methods, their characteristics, and their strengths and weaknesses were summarized.

Analyzing the previous research according to the dataset, image processing, extracted features, and ensemble learning processes, we find that our proposal exploits the classification features in detail and contains novelty by extracting them from images with a modified contrast. The captured fundus eye images may contain undesirable information, such as noise, which can be degraded by blur or, moreover, can have a low contrast. Thus, by improving the images, the quality of the features increased, and by extracting them from this type of image in our experiment, it was found that our proposal can influence, in a good sense, the accuracy classification.

The significant contributions of this work are as follows:

1. The images with the same structure were removed after checking their similarity with the structural similarity index (SSIM). The redundant information has been eliminated in order to obtain clean and non-repetitive data.
2. We designed adjustment parameters by contrast as gamma correction and creating new image sets for each DR level.
3. We computed Shannon and fuzzy entropies from all images.
4. We implemented a fully automatic ensemble learning ML framework applicable to DR diagnosis and binary classification between NoDR/Mild, NoDR/Moderate, NoDR/Proliferate, and NoDR/Severe classes and extracting base classifiers.
5. We developed the fastest, most accurate, and most reliable EL model for the DR level.
6. The bootstrap statistical technique is used to validate the relevant model.

## 3. Materials and Methods

### 3.1. Dealing with Duplicate Images

To prevent repetitive values in the dataset, a method was created for every DR level to detect similar images in the IDRiD dataset. In a loop, each image was compared with the other ones and, between them, the structural similarity index (SSIM) was applied, if, between two images, the SSIM was equal to 1, then the duplicate was removed.

By computing the SSIM index between gray-level images, color images with red, green, or blue color systems were transformed. The SSIM index of the two images was calculated only if the images had the same resolution. The number of images in our study was not high, but the model could have been less reliable if similar images were kept.

The SSIM index analyses the local brightness *l*(*x*,*y*), contrast *c*(*x*,*y*), and structure of both images *s*(*x*,*y*) [26].

(1) $$l\left(x,y\right)=\frac{2\mu_{x}\mu_{y}+C_{1}}{\mu_{x}^{2}+\mu_{y}^{2}+C_{1}},\;c\left(x,y\right)=\frac{2\sigma_{x}\sigma_{y}+C_{2}}{\sigma_{x}^{2}+\sigma_{y}^{2}+C_{2}}\;,\;s\left(x,y\right)=\frac{2\sigma_{xy}+C_{3}}{\sigma_{x}\sigma_{y}+C_{3}}$$

where μ is mean, σ is standard deviation, constant $C_{1}={\left(K_{1}L\right)}^{2}$, $C_{2}={\left(K_{2}L\right)}^{2},C_{3}=C_{2}/2$, $K_{1}\ll 1,\;K_{2}\ll 1$ ($K_{1}$ and $K_{2}$, usually are set to 0.01 and 0.03, respectively)

(2) $$\sigma_{xy}=\frac{1}{N-1}\sum_{i=1}^{N}\left(x_{i}-\mu_{x}\right)\left(y_{i}-\mu_{y}\right)$$

The components *l*(*x*,*y*), *c*(*x*,*y*), and *s*(*x*,*y*) are combined in the next expression weighted by with exponents α, β, and γ.

(3) $$SSIM\left(x,y\right)={\left[l\left(x,y\right)\right]}^{\alpha }\cdot {\left[c\left(x,y\right)\right]}^{\beta }\cdot {\left[s\left(x,y\right)\right]}^{\gamma }$$

### 3.2. Image Preprocessing and Feature Extractions

#### 3.2.1. Image Preprocessing

Enhancing the image is the goal of manipulating image contrast. Many algorithms have been developed to enhance the quality of medical images in pre-processing, as they are influenced by various types of artifacts. Due to the sensitivity of the human visual system to changes in luminance, brightness, or edges, this aspect is necessary. Linear or non-linear transformations can be utilized to modify contrast. The following proposed a non-linear transformation that used gamma correction.

The power function (Equation (4)) was applied on histogram having the *u*(*i*) gray-level $u=\left[u\left(0\right)\;u\left(1\right),\ldots ,u\left(N-1\right)\right]$, where *i* = 0, …, L − 1.

The non-linear power function *T*(*u*) with *γ* gamma correction parameter was expressed by the following:

(4) $$T\left(u\right)=\left(N-1\right){\left(\frac{u}{N-1}\right)}^{\gamma }$$

The contrast and tone of the image can be changed by modifying the γ exponent with the Lemmas 1 and 2, proposed by Rahman et al. [13].

**Lemma** **1.**  “*For low-contrast images, γ remains greater than 1*”.

**Lemma** **2.**  “*For high- or moderate-contrast images, γ∈[0.90,1.65]*”.

**Figure 1:**
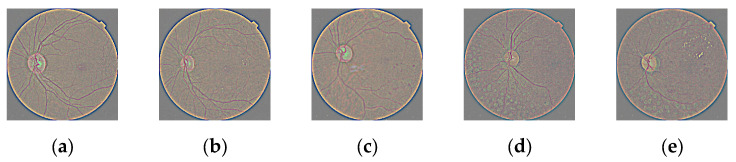
Sample fundus eyes images; (**a**) NoDR; (**b**) Mild; (**c**) Moderate; (**d**) Proliferate; (**e**) Severe.

**Figure 2:**
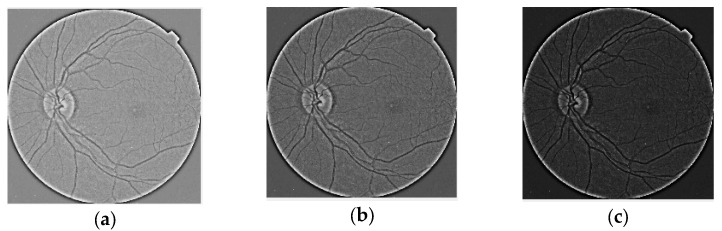
Image preprocessing stage results. (**a**) γ1 = 0.9; (**b**) γ2 = 1.65; (**c**) γ3 = 3.

Figure 1 shows the original images for each DR class from IDRiD dataset, and the examples of the contrast reversal transformation for γ1 = 0.9; γ2 = 1.65; γ3 = 3 are shown in Figure 2, image preprocessing stage results. (a) γ1 = 0.9; (b) γ2 = 1.65; (c); γ3 = 3.

#### 3.2.2. Feature Extraction

Extracting image features from histograms, patterns, textures, shapes, or fractals is possible. By analyzing the pixel arrangement, entropy plays an important role in pattern recognition from medical images, which allows for this feature to be successfully applied [27]. The features utilized in this study were derived from Shannon and fuzzy entropies [27,28].

The entropy provides information about the amount of randomness (or uncertainty) in an image based on the many shapes, textural features, or histogram features. When the content of an image changes depending on the DR levels, the values of entropy vary as a quantitative measure of the information contained in an image. Moreover, two distinct types of entropies were proposed, and DR levels were classified using them [29].

**Figure 3:**
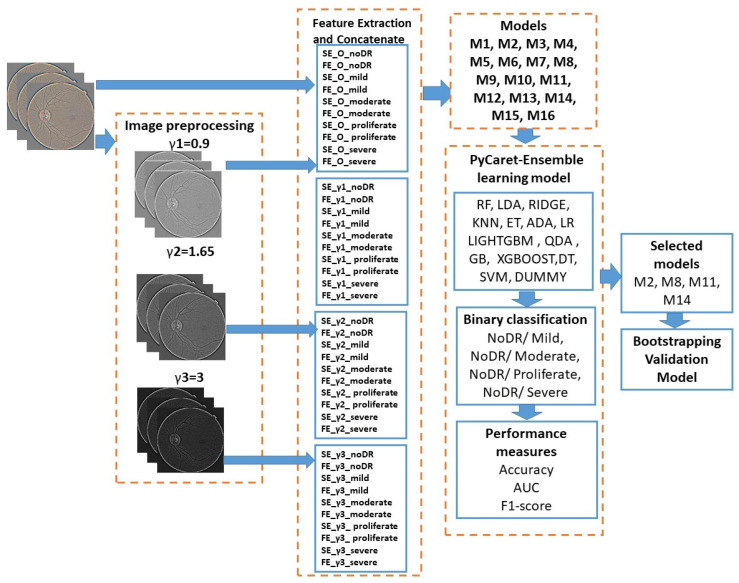
The general structure of the proposed workflow diagram.

These were computed for the manipulation of the contrast with the gamma correction of γ1 = 0.9; γ2 = 1.65; γ3 = 3, corroborating this value with entropy and DR levels. Ten features are summarized for each combination (see Figure 3).

(1) Shannon entropy (SE) [28]:

(5) $$H_{ShanEn}\left(x\right)=-\sum_{i=0}^{N-1}p_{i}\log p_{i}$$

(2) Fuzzy entropy (FE) [27,30]:

(6) $$H_{Fuzzy}=-\sum_{i=0}^{N-1}\frac{p_{i}\times \mu \left(p_{i}\right)}{\sum_{i=0}^{N-1}p_{i}\times \mu \left(p_{i}\right)}\times \ln\left(\frac{p_{i}\times \mu \left(p_{i}\right)}{\sum_{i=0}^{N-1}p_{i}\times \mu \left(p_{i}\right)}\right)$$

where *N* was the number of gray levels, *p_i_* was the probability associated with gray-level *i*, *μ* was the mean of the gray level, and *ε* was a positive threshold value with |*p_i_*| ≤ *ε*.

#### 3.2.3. AutoML with PyCaret

PyCaret is a simple and efficient AutoML that improves the efficiency of EL and accelerates research on ML.

As an automatically trained program, the data PyCaret (3.0.4 version) was selected, against some of the open-source alternatives, such as H2O AutoML, AutoWEKA, Auto-PyTorch, Auto-sklearn, etc. Usually, the AutoML technique automates machine learning workflows for binary classification, detection, or prediction processes. In our study, PyCaret combines the best ensemble models, such as Naive Bayes (NB), random forest classifier (RF), linear discriminant analysis (LDA), ridge classifier (RIDGE), K-neighbors classifier (KNN), extra trees classifier (ET), Ada boost classifier (ADA), logistic regression (LR), light gradient boosting machine (LIGHTGBM), quadratic discriminant analysis (QDA), gradient boosting classifier (GBC), extreme gradient boosting (XGBOOST), decision tree classifier (DT), SVM—linear kernel (SVM), and dummy classifier (DUMMY) classifiers.

After removing the similar images from the remaining 99%, the features obtained from these were divided into the 70% training set and 30% testing set to ensure that the model was trained on diverse and sufficient data.

The results of the classification are displayed in Section 4, where PyCaret was used to apply various classifiers, such as NoDR/mild, NoDR/moderate, NoDR/proliferate, and NoDR/severe.

### 3.3. Proposed Methodology

This paper sought to experiment with ensemble learning (EL) on a dataset that was constructed from Shannon (SE) and fuzzy entropy (FE) and computed using contrast manipulation. The preprocessing and evaluation experiments were conducted using Matlab2018a and Python (3.9 version) programming languages as well as libraries such as Image Processing and PyCaret (3.0.4 version). The Indian Diabetic Retinopathy Image Dataset (IDRiD) was proposed [31]. It contains color fundus images of NoDR (1805), mild (370), moderate (999), proliferate (295), and severe (293) cases, and the number of images is provided in brackets. The images from Retinal Fundus Camera Model: Kowa VX-10α were acquisitioned; these belonged to the Eye Clinic, Sushrusha Hospital Building, Nanded (M.S.), India and are publicly available.

The hardware environment had the following architecture: Processor Intel(R) Core(TM) i7-1065G7 CPU @1.30–1.50 GHz, RAM 16.0 GB, and Windows 11 operating system, 64-bit, x64-based processor.

A workflow diagram is shown in Figure 3, which describes our methodology. The general content is divided into four main blocks.

- Images were processed before manipulating contrast.
- Ten features were obtained for each image set after extracting the features. Four subblocks were created by computing these for each level of contrast, type of entropy, and level of DR. Table 2 stores the 16 models and features that were contained; these were extracted from the four subblocks, and in each model, we selected the features extracted from noDR and each level of DR disease.
- Fifteen MLs were fed with the features proposed in Table 2 for the ensemble learning process, which was performed with the PyCaret tool. After, extraction of the features, four groups of models occurred, as in Table 3. In terms of accuracy, the AUC and F1 score metrics were evaluated for binary classification (see Table 4).
- The last block consisted of an evaluation of each selected model (see Table 5) in the previous step with the bootstrapping statistical technique. In this sense, 100 subsets were generated, and these became new training datasets. Each new training dataset picked a sample of observations with a replacement from the original dataset; in this way, each selected classifier shown in bold in Table 5 was retrained 100 times for each subset, and the average of the generated accuracy across 100 bootstrap samples of the held-out test set was stored in order to validate the model. The best classifier was chosen based on the accuracy, area under the curve, and F1 score, and their connections are presented in Table 6.

The ambition of our work was to find a possible model composed of relevant features and train an AutoML for refining and classifying DR levels, thereby making meaningful contributions to both the healthcare sector and the field of ML.

For an easy understanding, in the feature extraction block, the name of the features had the following interpretation: SE and FE were the names of entropy; letter O meant unprocessed images; γ1, γ2, and γ3 were gamma correction indexes; and the last part of the feature’s name was on the DR level.

**Table 2:** The content of each model and the attached features.

Model Index	Features
M1	SE_O_noDR. SE_O_mild, FE_O_noDR, FE_O_mild
M2	SE_γ1_noDR, SE_γ1_mild, FE_γ1_noDR, FE_γ1_mild
M3	SE_γ2_noDR, SE_γ2_mild, FE_γ2_noDR, FE_γ2_mild
M4	SE_γ3_noDR, FE_γ3_noDR, SE_γ3_mild, FE_γ3_mild
M5	SE_O_noDR, SE_O_proliferate, FE_O_noDR, FE_O_proliferate
M6	SE_γ1_noDR, SE_γ1_proliferate, FE_γ1_noDR, FE_γ1_proliferate
M7	SE_γ2_noDR, SE_γ2_proliferate, FE_γ2_noDR, FE_γ2_proliferate
M8	SE_γ3_noDR, SE_γ3_proliferate, FE_γ3_noDR, FE_γ3_proliferate
M9	SE_O_noDR, SE_O_severe, FE_O_noDR, FE_O_severe
M10	SE_γ1_noDR, SE_γ1_severe, FE_γ1_noDR, FE_γ1_severe
M11	SE_γ2_noDR, SE_γ2_severe, FE_γ2_noDR, FE_γ2_severe
M12	SE_γ3_noDR, SE_γ3_severe, FE_γ3_noDR, FE_γ3_severe
M13	SE_O_noDR, FE_O_noDR, SE_O_moderate, FE_O_moderate
M14	SE_γ1_noDR, SE_γ1_moderate, FE_γ1_noDR, FE_γ1_moderate
M15	SE_γ2_noDR, SE_γ2_moderate, FE_γ2_noDR, FE_γ2_moderate
M16	SE_γ3_noDR, SE_γ3_moderate, FE_γ3_noDR, FE_γ3_moderate

**Table 3:** Grouping of models in terms of DR and preprocessing levels.

Model Groups	Grouping Explanation
M1, M5, M9, M13	Models are tested with features extracted from original images
M2, M6, M10, M14	Models are tested with features extracted from images preprocessed with γ1
M3, M7, M11, M15	Models are tested with features extracted from images preprocessed with γ2
M4, M7, M12, M16	Models are tested with features extracted from images preprocessed with γ3

**Table 4:** Classification performance measures.

Metrics	Explanations	Equations (True Positives (TP), the False Positives (FP), the True Negatives (TN) and the False Negatives (FN))
Accuracy (ACC)	It shows how well the model correctly classified the different classes [32].	$ACC=\frac{TP+TN}{TP+TN+FP+FN}$
Area Under the Curve (AUC)	AUC is a measure of the performance of an estimator in binary classification problems [32].	$AUC=1-\frac{1}{2}\left(\frac{FP}{FP+TN}+\frac{FN}{FN+TP}\right)$
F1 score	F1 score is computed with precision and recall, and it evaluates proposed method [32].	$F1-score=\frac{2\times precision\times recall}{precision+recall}$ $precision=\frac{TP}{TP+FP}$ $recall=\frac{TP}{TP+FN}$

**Table 5:** Tune hyperparameters for classification ML algorithms for each model.

Model Index	Classifier	Hyperparameters
M1	LGBM	boosting_type = ‘gbdt’, learning_rate = 0.1, num_leaves = 31
M2	XGB	booster = ‘gbtree’, n_estimators = 100,
M3	RF	criterion = ‘gini’, n_estimators = 100
M4	GBC	criterion = ‘friedman_mse’, n_estimators = 100, random_state = 123,
M5	XGB	booster = ‘gbtree’, n_estimators = 100,
M6	XGB	booster = ‘gbtree’, n_estimators = 100,
M7	XGB	booster = ‘gbtree’ n_estimators = 100
M8	RF	criterion = ‘gini’, n_estimators = 100
M9	XGB	booster = ‘gbtree’ n_estimators = 100
M10	LIGHTGBM	boosting_type = ‘gbdt’, n_estimators = 100, num_leaves = 31,
**M11**	**GBC**	**criterion = ‘friedman_mse’, n_estimators = 100,** **random_state = 123,**
M12	KNN	algorithm = ‘ auto’, leaf_size = 30, metric = ‘minkowski’, n_neighbors = 5
M13	XGB	Booster = ‘gbtree’ n_estimators = 100
M14	**LIGHTGBM**	**boosting_type = ‘gbdt’, n_estimators = 100, num_leaves = 31,**
M15	RF	criterion = ‘gini, n_estimators = 100,
M16	KNN	algorithm = ‘auto’, leaf_size = 30, metric = ‘minkowski’, n_neighbors = 5

**Table 6:** Performance ensemble learning of the proposed method (5-fold CV).

Classes	Model	Accuracy	AUC	F1_Score
No_DR/moderate	M1	0.870	0.938	0.899
	**M2**	**0.882**	**0.946**	**0.909**
	M3	0.880	0.942	0.906
	M4	0.880	0.942	0.907
No_DR/proliferate	M5	0.917	0.927	0.683
	M6	0.917	0.937	0.686
	M7	0.918	0.927	0.680
	**M8**	**0.922**	**0.953**	**0.702**
No_DR/severe	M9	0.916	0.924	0.510
	M10	0.920	0.929	0.537
	**M11**	**0.929**	**0.941**	**0.902**
	M12	0.919	0.910	0.490
No_DR/mild	M13	0.916	0.949	0.746
	**M14**	**0.925**	**0.942**	**0.779**
	M15	0.928	0.934	0.780
	M16	0.918	0.947	0.757

PyCaret tool has included a confusion matrix (CM) and various performance metrics extracted from the CM, with the aim of comparing the various MLs. The accuracy, AUC and F1 score are indispensable metrics, which were used to evaluate the performance of the AutoML. The mathematic approaches and definition of the metrics are provided in Table 4. Through exploration of fifteen classifiers, we intended to delineate the adequate model, which was meant to be a step forward in combating this disease.

## 4. Results and Discussion

For experimentation purposes, the PyCaret ensemble learning tools were integrated. This choice was made because it is a powerful machine learning technique that combines multiple classifiers in order to choose only one with a higher accurate prediction and optimum hyperparameters. Our model is compared with other ML models in terms of metrics extracted from the confusion matrix.

Before selecting a final model, a total of 16 models are tested. The ensemble models were tested using 5-fold cross-validation, with data being split into 70:30 training/test data.

The input features, selected classifiers from PyCaret, and important tuned hyperparameters are stored in Table 5. It should be mentioned that the features were chosen to identify the same DR level. The concatenate steps consist of grouping features into 16 models.

The XGB classifier was the most selected, which was six times (M2, M5, M6, M7, M9, and M13 models), with hyperparameters and the boosters based on tree models controlled by 100 trees. The next one was RF, and it was selected three times (M3, M8, and M15 models) with the same number of trees, and for measuring the quality of a split, the Gini criterion was chosen. The GBC (M11 and M13 models), LIGHTGBM (M10, M14 models), and KNN (M12 and M16 models) classifiers were selected twice, the GBC and LIGHTGBM having the same number of estimators, but the criteria of data selection were different. The search for neighbors was performed using the KNN classifier with five neighbouring numbers and a Minkowski distance metric.

In our experiment, four groups of models were obtained according to gamma correction and both entropies. Each group is shown in Table 3. Table 6 displays the binary classification obtained from confusion matrices resulting from both Shannon and fuzzy entropies.

The values of the metrics for all these models that include the original images are smaller than the values obtained from the models with modified gamma correction according to lemma 1 and 2. It highlights for each DR level, the class combination, the selected models, and the classifier, so the combinations No_DR/ moderate, M2 and XGB; No_DR/ proliferate, M8 and RF; No_DR/ severe, M11 and GBC; and No_DR/ mild, M14 and LIGHTGBM, were obtained.

For the M2, M8, M11, and M14 models, the bootstrapping statistical technique was applied to the training set and run for 100 iterations. The model was validated in terms of the accuracy score. The results indicate that the values M2 (0.896), M8 (0.931), M11 (0.937), and M14 (0.932) were observed. The selected models are accurate and robust. Both 5-fold CV and bootstrapping maintained a high accuracy.

In our experiment, the combination between features SE_γ2_noDR, SE_γ2_severe, FE_γ2_noDR, FE_γ2_severe, and the GBC classifier provided the best results. The accuracy was 0.929, the AUC was 0.941, and the method was validated by F1 with a score of 0.902.

The algorithm embedded into the GBC classifier is designed to build an additive model in a forward stage-wise fashion. This solution is capable of optimizing arbitrary differentiable loss functions and can effectively categorize the extracted data stored in the M11 model. The best decision was made by this model and GBC.

Comparing our results with the scientific literature that deals with EL and various databases, we stored the methods, datasets, and metrics in Table 7.

The IDRiD dataset was selected in order to detect the best model because it has a large number of samples, it is the largest publicly available database, and the images are already classified by physicians. Our study’s main limitation is that the images are not segmented into regions of interest, and the features are calculated from the entire image.

Recently, a new trend has appeared in the classification of DR levels; the state-of-the-art studies show the application of ensemble learning, and it became an efficient tool that can be included in the current trends. The general problem that is addressed is if the preprocessing can influence the classification process; with this study, we know the answers of this issue. In the empirical process, we determined that the manipulation of the contrast can influence the entropy values and, finally, the classification.

In this paragraph, some limitations are highlighted. A greater number of features can influence the classification; furthermore, their structure, such as color, shapes, or edges, is also a fact that can be taken into account in the future.

## 5. Conclusions

In this work, we applied EML to features extracted from the original and modified images with the contrast modified by γ1 = 0.9; γ2 = 1.65, and γ3 = 3. The Shannon and fuzzy entropy features were successfully utilized in DR-level classifications, with the most accurate result being 0.929. Hyper-tuning various classifiers and selecting the best classifier are necessary to validate the results in the context of EL. The M11 model was the optimal choice for the proposed input data, as it confirms the features of SE_γ2_noDR, SE_γ2_severe FE_γ2_noDR, FE_γ2_severe features. Based on the experimental results, it can be concluded that the base GBC classifier was chosen for classes No_DR/severe. Future trends will suggest using features extracted from the pattern texture of medical images and ensemble learning tools to classify the original data.

## Figures and Tables

**Table 1:** Strengths and weaknesses of relevant papers.

References	Method	Strengths	Weakness
[1]	EL	Detecting duplicate images and removing them.	Has moderate accuracy and F-measure performance
[12]	KNN	Hyperparameters optimization is employed to tune	The performance is not effective.
[13]	Neural Network SVM	Segmentation of blood vessels in RD is performed.	The hyperparameters are not tuned The performance is not good enough for the accuracy.
[17]	CNN	Pre-trained convolutional neural networks is applied	CNNs are very time-consuming
[19]	EL	Selecting the important features and EL	The performance is not good enough for the accuracy.
[20]	EL	Emphasizes features extracted from anatomical components	The rest and training dataset do not contain the same preprocessing methods
[21]	EL	Proposed method is fully automatic using a bagging ensemble learning technique	Computationally expensive
[22]	NB, KNN, SVM, MP, RF, LR	These data were fed into a novel Modified Moth Flame Optimization-based feature selection	The hyperparameters are not tuned.

**Table 7:** A comparison table of the state-of-the-art approaches.

Reference and Year	Method	Dataset	Metrics
Porwal et al., 2018 [31].	EL	SJRUH	74.49 accuracy
Sabbir et al., 2020 [33].	EL	MESSIDOR	92.0% accuracy
Odeh et al., 2021 [34].	EL	Messidor (InfoGainEval.)	70.7% accuracy
Du et al., 2022 [35].	EL	DiaretDB1	79.3% AUC
Luo et al., 2023 [36].	CNNs	EyePACS datasets	92.1% accuracy 96.7% AUC
Alshayeji et al., 2023 [23].	EL (Boosted trees)	Kaggle EyePACS datasets	91.7% accuracy
Ours (M11 model)	EL	IDRiD	92.9% accuracy, 94.1% AUC 90.2% F1 score

## Data Availability

Data are contained within the article.
